# Cultural Competence of European Nursing Faculty. An International Cross‐Sectional Study

**DOI:** 10.1111/jnu.70000

**Published:** 2025-03-16

**Authors:** Laura Visiers‐Jiménez, María Isabel Baeza‐Monedero, José Ríos‐Díaz, Sylvain Marcel Lybrecht Llinares, Maria Lara Martínez‐Gimeno, Karel Mattys, Rudy de Sagher, Ria Bruijn, Sybille Frey, Martina Valachovičová, Kathrine Hoffmann Pii, Lisbeth Vinberg, Janne Krag, Klaus Muller, Gordon Hill, Maria Malliarou, Angela Flynn, Federica Canzan, Raffaello Furlan, Beatrice Mazoleni, Ilaria Fava, Giuseppina Tomaiuolo, Agita Melbārde‐Kelmere, Cristina Pestaña, Ermira Tartari Bonnici, Josef Trepani, Pascale Didry, Mirjam Palsma, Andreia Lima, Teresa Moreira, Jose Edmundo Xavier Furtado Sousa, Claudia Baccatum, Carlos Sequeira, Jose Carlos Carvalho, Rita Mónaco, Emina Hadziabdic, Aynur Uysal Toraman, Viktorija Kiele, Pedro Ventura Puertos, Adelina Martín Salvador, María Adelaida Álvarez, María Gázquez López, María Angustias Sánchez Ojeda, Mª Angeles Pérez Morente, Shakira Kaknani, Juan Carlos Paramio Cuevas, Rocío Romero Serrano, Ana Fernández Feito, Fernando Alonso Pérez, Juan Carlos Fernández Domínguez, Alfonso M. García Hernández, Jose Luis Cobo Sánchez, Esmeralda Santa Cruz, Raúl Soto Cámara, Rubén García Fernández, Elena Fernández Martínez, María Hinojal Benavente Cuesta, Rosa María Cárdaba García, Silvia Costa Abos, Nuria Gorchs Font, Mª del Carmen Malagón Aguilera, José Tomás Mateo, Francesc Valls Fonayet, Agueda Cervera Gasch, Mª Zoraida Clavijo Chamorro, Adela Gómez Luque, Cristina Jorge Soto, Elena Andrade Gómez, Gema Cid Expósito, Raquel Luengo González, Isabel Font Jiménez, Beatriz Álvarez Embarba, Sonsoles Hernández Iglesias, Elena Jiménez García, Alonso Molina Rodríguez, Mª Dolores Pereñiguez Olmo, Maider Belintxon Martín, Saloa Unanue Arza, Maria Isabel Trespaderne Beracierto, Elena de Lorenzo Urien, Cristina Lozano Ochoa, José Ríos Díaz, Sylvain Marcel Lybrecht Linares, María Lara Martínez Gimeno, María Isabel Baeza Monedero, Laura Visiers Jiménez

**Affiliations:** ^1^ San Juan de Dios Foundation Madrid Spain; ^2^ Health Sciences Department Comillas Pontifical University San Juan de Dios School of Nursing and Physical Therapy Madrid Spain; ^3^ SALBIS Research Group, Faculty of Health Sciences University of Leon Ponferrada Spain

**Keywords:** cultural competence, education, faculty, health educators, nursing, nursing, transcultural nursing

## Abstract

**Introduction:**

The diverse cultural landscape of Europe underscores the importance of culturally safe healthcare. There is a necessity to assess cultural competence among European nursing faculty to provide an international perspective on cultural competence.

**Design:**

A descriptive, cross‐sectional study.

**Methods:**

An assessment of cultural competencies was conducted using the Cultural Competence Assessment scale, either in its original language (English) or in its translated and validated versions in Spanish, Italian, Portuguese, and Turkish. An online questionnaire was used to collect data.

The study was conducted in 71 higher institutions, distributed across 17 countries through a consecutive sample of 1364 nursing faculty.

The ethical principles of biomedical research were respected during the study, and the confidentiality of the data was guaranteed.

**Results:**

The mean level of cultural competence of the European nursing faculty was at the level of ‘good’. They showed greater cultural awareness and sensitivity than cultural competence behaviors. Significant associations were found between cultural competence level and the language of the questionnaire, level of education, having a nursing degree, leisure stays abroad, having friends from other countries or cultures, and international experiences abroad and at home. The better levels of cultural competence were found in profiles with the categories of: women with a Nursing Degree, a higher level of education, and with an ERASMUS+ stay experience.

**Conclusions:**

This study offers an international overview of the cultural competence of nursing faculty. While the overall level of cultural competence was good, there is a need to reinforce the behaviors and factors that influence it.

## 1 Introduction

The International Council of Nurses (ICN), representing millions of nurses worldwide, states that nurses must be culturally competent to provide appropriate and effective care to patients regardless of their cultural background (ICN 2013). Therefore, nursing education should ideally promote respect for fundamental rights and work against all forms of discrimination in healthcare settings, so that nursing students can positively interact with peers and patients who are culturally different (Hagqvist et al. 2020).

Nevertheless, literature indicates that this assumption is yet to be a reality. Different levels of teaching and training quality have been addressed in various studies reporting that nursing faculty are not prepared to teach cultural content (Farber 2019; Kaihlanen et al. 2019). But, at the same time, nursing faculty have been appointed as key reference figures in facilitating cultural competence (CC) development in their students (Flood and Commendador 2016; Gradellini et al. 2023; Shin et al. 2016). In fact, it has been acknowledged that low levels of cultural competence among nursing faculty are usually linked to students graduating from nursing programs with low levels of cultural competence as well (Baghdadi and Ismaile 2018). Paradoxically, there are few studies focusing on the intercultural competence level of educators (Kirby et al. 2021; De‐María et al. 2024).

Consequently, studying whether European nursing faculty members are culturally competent becomes the core focus of this research. With the aim of describing the competence level of nursing faculty across different European nursing higher education institutions, two questions are raised: (1) what is the level of cultural competence of nursing faculty? and (2) which background factors are associated with the level of cultural competence of nursing faculty?

Europe is comprised of multiple countries where 24 different official languages, and at least five religions coexist (European Union 2023). In this challenging melting pot, cultural differences in terms of ethnicity, religion and cultural background unlock different understandings of what constitutes health or illness and how illness should be handled (Antón‐Solanas, Tambo‐Lizalde, et al. 2021), highlighting the need for healthcare programmes that meet the expectations and needs of every individual and reduce disparities in quality healthcare (Antón‐Solanas, Tambo‐Lizalde, et al. 2021; Parić et al. 2021).

Focusing on healthcare, it has been shown that training healthcare professionals in intercultural skills can help reduce healthcare inequity. Some of the benefits of providing culturally competent care have been associated with an improved quality of care and patient satisfaction, positive health outcomes for patients, improved nurse–patient communication, and fewer racial and ethnic differences in healthcare, which, in short, means providing a better health status (Antón‐Solanas, Tambo‐Lizalde, et al. 2021; Yılmaz et al. 2017). In contrast, failure to adapt care to a culturally diverse population may lead to healthcare inequalities, lack of cultural safety for patients (i.e., the most effective nursing practices for people from another culture, taking into account and accepting cultural differences), misinterpretation of patients' needs, dissatisfaction with care, inaccurate diagnoses and treatment errors, adverse health outcomes, prolonged length of stays, avoidable hospitalizations, under‐ and over‐utilization of procedures, etc. (Antón‐Solanas, Huércanos‐Esparza, et al. 2021; Smallwood 2018; Vogel 2014).

In this context, efforts to improve clinical cultural competence to better prepare students to enter the healthcare and social services fields as clinically competent professionals have dominated the discussion over the last few decades (Flaubert et al. 2021).

The conception and operationalization of cultural competence models and the implementation of interventions for enhancing it in healthcare professionals are heterogeneous in Europe. A systematic scoping review of the empirical publications focused on this topic included mainly references to the Campinha‐Bacote model, the Betancourt model, and the Papadopoulos one among others (De‐María et al. 2024). The most used models for delivering cultural competence interventions in healthcare are based on Leininger's Theory of Transcultural Nursing Care, defining a culturally competent professional capable of assessing and understanding culture, care, and health factors and using this knowledge creatively with people of diverse or similar lifeways (Leininger and McFarland 2002).

According to Doorenbos et al. (2005), Cultural Competence encompasses the development of cultural diversity experiences, cultural awareness (knowledge), adoption of culturally sensitive attitudes, and the demonstration of culturally competent behavior in everyday practices. Schim and Doorenbos (2010) describe, in their Three‐Dimensional Model of Cultural Congruence, the concepts of cultural awareness, sensitivity, and behavior. Cultural Awareness is a cognitive construct that involves knowledge and thought to appreciate the ways in which cultures vary and how cultural contexts influence personal meaning. Cultural sensitivity is an affective construct that involves clinicians' attitudes about themselves and others, their openness to learning about cultural dimensions and diversity, and self‐exploration of personal cultural heritage and experiences. Cultural competence behaviors are actions in response to the demands of cultural diversity, being aware and sensible of the differences and barriers that may occur when people of diverse cultures interact and communicate (Schim and Doorenbos 2010).

All these frameworks used in previous studies underscore the complexity of culture as a multifaceted and fluid construct, which is particularly relevant in the context of educating nurses.

Cultural humility has emerged as a vital concept in healthcare, offering a complementary perspective to cultural competence. While cultural competence emphasizes the development of specific skills and knowledge about diverse cultures, cultural humility focuses on an ongoing process of self‐reflection, lifelong learning, and addressing power imbalances inherent in healthcare interactions. Together, these concepts highlight the complexity of fostering culturally responsive care through nursing education, requiring both structural and personal efforts (Tervalon and Murray‐García 1998; Foronda et al. 2016). In this regard, recent literature has been published about state‐of‐the‐art tools (such as mobile apps) specifically designed to boost the cultural capacity and humility of nursing students (Farsangi et al. 2023).

Therefore, the challenge lies in whether universities can bring the issue of culture to the forefront of current academic needs, as teaching cultural awareness in nursing education can present a major professional challenge for nursing educators (Oikarainen et al. 2017). Guidelines for implementing cultural content in the nursing curricula have been made in the United States as well as in Europe (Sairanen et al. 2013), and they clearly state that nursing programs should be reviewed to establish how cultural content is integrated throughout each curriculum.

In this sense, university curricula may include a wide variety of activities that could be somehow related to boosting cultural competence, such as internationalization at home activities, international volunteering programs, service‐learning activities, etc. Among the various alternative activities that universities offer to enhance intercultural competence, ERASMUS+ stands out as the most common. This program, which supports education, training, youth, and sports across Europe, is available at every university on the continent. The 2021–2027 program places a strong focus on social inclusion, the green and digital transitions, and promoting young people's participation in democratic life.

Still, this lack of cultural competence knowledge is not an issue purely limited to higher education environments, but also affects nursing clinical practice, where students spend a high number of hours during their college training. A recently published study found that clinical mentors identified the need to promote a more culture‐sensitive evaluation process among students, but they also recognized two major setbacks: firstly, the lack of resources to do that and, secondly, their own need to update their cultural competence (Hagqvist et al. 2020).

Thus, preparing future nurses to provide culturally competent nursing care requires significant education and training and should be a core component of nursing education (Antón‐Solanas, Huércanos‐Esparza, et al. 2021).

According to all the evidence presented above, it seems to be clear that further studies are needed to explore the actual level of cultural competence of nursing faculty (Kirby et al. 2021) which would constitute the starting point for designing active strategies to prepare culturally competent nurse educators within higher education environments. The aim of the present study is, therefore, to describe the cultural competence level of nursing faculty from European nursing higher institutions participating in the CCA‐EUnurse (Cultural Competence Assessment in the Nursing Degree within the European Higher Education Space) project.

## 2 Methods

### 2.1 Design

Descriptive, multicenter, cross‐sectional design. The study was conducted in 17 European countries among 71 higher education institutions that are part of the CCA‐EUnurse consortium.

### 2.2 Participants

Inclusion criteria for taking part in the study comprised: nursing faculty who provided theoretical and/or practical training for students in the Bachelor's Degree in Nursing during the academic year 2021–2022 and who also agreed to participate voluntarily in the study by signing the informed consent form.

The target population for the study was 4284 nursing faculty. A consecutive nonprobabilistic sampling was carried out, and 1354 nursing faculty were recruited from across all the participating nursing higher education institutions.

### 2.3 Data Collection

Individual self‐perception regarding cultural competence was measured with the Cultural Competence Assessment Scale (CCA), developed to assess CC across a broad range of disciplines and educational levels, in its original version in English (Doorenbos et al. 2003, 2005) and its translated, adapted, and validated versions in Spanish (CCA‐S) (Raigal‐Aran et al. 2019), Italian (CCA‐I) (Caricati et al. 2015), Portuguese (CCA‐P) (Raigal‐Aran et al. 2023), and Turkish (CCA‐TR) (Uysal Toraman et al. 2023). All of them presented good content validity, acceptable internal consistency, and adequate construct validity. Table 1 summarizes the properties of each version of the questionnaire. Every scale has been commonly divided into two dimensions: Cultural Awareness and Sensitivity and Cultural Competence Behavior. A higher score in the scale indicates a greater cultural competence. Since the scale has a slightly different number of items, depending on the language, the final score was normalized from 0 to 100 in order to compare between versions. Also, for a better interpretation of the results, the definition of the competence level was divided into four categories, as suggested by Kajander‐Unkuri et al. (2021) when measuring the self‐assessed level of competences, based on the Three‐Dimensional Model of Cultural Congruence and adapted from Luquis and Pérez (2005) description: Low level or cultural incompetence (≤ 25): poor cultural awareness, sensitivity, and behavior requiring significant guidance and support in culturally nursing practice. An individual who lacks an understanding of the differences among racial, ethnic, and cultural groups and is at the lowest level of the process of cultural competence. Rather good level or cultural awareness (26–50): An individual who is sensitive to the values, beliefs, and practices of different racial, ethnic, and cultural groups but still needs guidance and support. This might be seen as acknowledging basic aspects of a patient's cultural background but lacking deeper understanding and application; Good level (51–75): An individual who is culturally sensitive to the needs of different racial, ethnic, and cultural groups and is able to respond appropriately to the needs of these groups in culturally diverse situations. They can apply their cultural knowledge in planning and delivering patient care, which enhances the quality of care and patient experience; Very good level or cultural proficiency (76–100): the individual is highly skilled and exemplary in culturally sensitive nursing practice. They can mentor others and develop care practices that comprehensively address the cultural needs of patients.

**TABLE 1:** Psychometrics of multilingual validated versions of the CCA.

Questionnaire	Authors, year	Version	Psychometrics	Number of items	Dimensions	Direct score	Normalized score
English (USA)	Schim et al. 2003	Original *n* = 117	Construct validity: 0.40 Concurrent validity with IAPCC: 0.67 Reliability (Cronbach's alpha): 0.92 Internal consistency CAS: 0.75 Internal consistency CCB: 0.93	25 items Likert (1 strongly agree/always to 7 not agree/never and option to “no response”) Items 1, 2, 5, 8 inverse scored	CAS: 11 items (1–11) CCB: 14 items (12–25)	25 to 175	0 to 100 (direct score/175)
Italian	Caricati et al. 2015	Adaptation *n* = 289	Construct validity: Confirmatory factor analysis Reliability (Cronbach's alpha): — Internal consistency CAS: 0.86 Internal consistency CCB: 0.78	26 items Likert	CAS: 10 items (1–11) Deleted item 9 CCB: 16 items (11–26) Added items 25–26	26 to 182	0 to 100 (direct score/182)
Spanish	Raigal‐Aran et al. 2019	Adaptation *n* = 568	Content validity: Delphi 16 experts IVC: 1 Construct validity: CFA Reliability (Cronbach's alpha): 0.86 Internal consistency CAS: 0.87 Internal consistency CCB: 0.86	25 items Likert (1 strongly agree/always to 7 not agree/never and option to “no response”) Items 1, 2, 5, 8 inverse scored	CAS: 11 items (1–11) CCB: 14 items (12–25)	25 to 175	0 to 100 (direct score/175)
Portuguese	Raigal‐Aran et al. 2023	Adaptation *n* = 284	Construct validity: CFA Reliability (Cronbach's alpha): 0.84 Internal consistency CAS: 0.70 Internal consistency CCB: 0.79	24 items Likert (1 strongly agree/always to 7 not agree/never and option to “no response”) Items 1, 2, 5, 8 inverse scored	CAS: 10 items (1–11) Deleted item 9 CCB: 14 items (11–26)	24 to 168	0 to 100 (direct score/168)
Turkish	Uysal Toraman et al. 2023	Adaptation *n* = 460	Content validity: Delphi 8 experts IVC: 0.94 Construct validity: CFA Reliability (Cronbach's alpha): 0.83 Internal consistency CAS: 0.96 Internal consistency CCB: 0.87	24 items Likert (1 strongly agree/always to 7 not agree/never and option to “no response”) Items 1, 2, 5, 8 inverse scored	CAS: 11 items (1–11) CCB: 14 items (12–25)	25 to 175	0 to 100 (direct score/175)

Abbreviations: CAS, Cultural Awareness and Sensitivity dimension; CCA, Cultural Competence Assessment; CCB, Cultural Competence Behavior dimension; CFA, confirmatory factor analysis; IAPCC, Inventory for Assessing the Process of Cultural Competence among Health care Professionals (IAPCC); IVC, Index Validity Content.

The measured sociodemographic and background variables before working life as a faculty member included: age, gender, religious community with which they most identified with, years of experience as a faculty, European country where they were teaching, higher education institution, level of education, possession of a nursing degree, previous professional experience in a healthcare context, and years of experience as a healthcare provider, mother tongue/native language, number of other languages spoken, leisure/study/working time abroad before working life as a faculty member, and having friends from other countries/cultures. The measured acquired factors after starting to work at their university were: ERASMUS+ experience as faculty in higher education, and participation in international activities taking place “at home”, in their own campus.

Data collection was performed from February to July 2022, through an online questionnaire (REDCap tool). The study was presented by the contact person (s) at each participating Higher Education Institution to its faculty. The presentation included a QR code with the link to the questionnaire. In addition, two reminders were sent by email 15 days and 1 month after the first contact.

### 2.4 Data Analysis

A database was created and exported into an Excel sheet (Microsoft Office 365 ProPlus 2016) to refine the data and detect outliers. The analyses were carried out with JASP 0.18 (JASP Team 2023) and R software v.4.3.1 for Windows.

Data were summarized by mean and standard deviation, quartiles, and range for continuous variables, and absolute and relative frequencies for categorical variables. The sample size allows us to assume normality in distributions.

One‐way analysis of variance (ANOVA) was used to examine the association between the background and internal factors and CAS, CCB, and CCA scores. The effect sizes were evaluated with *w* ^2^ (*w* ^2^ ≈ 0.02 small, medium ≈ 0.15 and large ≈ 0.35) (Lakens 2013).

In addition, multivariate linear regression models were performed to explore the profile of nursing faculty with higher cultural competencies. Beta standardized and unstandardized beta coefficients were calculated, and *R* and *R* ^2^% were obtained to evaluate the explicative power of the model.

For all tests, significant differences were assumed at *p* < 0.05 for a 95% confidence interval.

### 2.5 Ethical Considerations

The study protocol was approved by the Saint John of God Research Commission (Protocol No. P_2021_003). It was also reviewed and approved by each participating higher education institution. Before data collection, a full disclosure of the participants' rights, the nature and risks of the study, the benefits of the study, and voluntary participation forms were provided to the respondents. The researchers from each collaborating institution coordinated it. Online informed consent was secured from the respondents as a previous step to access the page of the questionnaire. Confidentiality was guaranteed throughout the research process.

## 3 Results

One thousand three hundred and sixty‐four nursing faculty (response rate = 32% of 4284) from 71 higher education institutions across 17 countries participated in the study (Table S1).

The frequency and the response rate of each item of the CCA scale in every language are presented in Table S2.

### 3.1 Demographics of Nursing Faculty

The mean age of nursing faculty was 46.4 (SD 10.44; range 20.5–74.5) years old, with a mean of 12.7 (SD 10.47; range 0–51) years of experience as faculty. 70% were female, 72% had a nursing degree, and 50% had a PhD level of education.

85% of nursing faculty had previous professional experience in healthcare contexts, with an average of 16.4 (SD 10.78; range 0–47) years of experience as a healthcare provider. 85.4% of them spoke at least one language in addition to their mother tongue.

Before their working life as a lecturer at university, 66% of the faculty had spent leisure time abroad, 27% study time abroad, and 22% working time abroad. Nevertheless, most faculty (82%) had never had an ERASMUS+ experience in university, while 41% of them had participated in other international activities “at home” (Table 2).

**TABLE 2:** Demographics of nursing faculty.

Variables	Categories	*n*	Percentage (%)
Background factors			
Gender	Female	950	69.6
	Male	407	29.8
	I prefer not to answer	7	0.5
Religous community	Christian	908	66.6
	Muslim	47	3.4
	Budist	5	0.4
	Other	10	0.7
	I do not identify with any option	360	26.4
	I prefer not to answer	34	2.5
Level of education	Graduate/Bachelor	197	14.4
	Postgraduate	488	35.8
	Doctorate	679	49.8
Nursing Degree	Yes	987	72.4
	No	377	27.6
Previous professional experience (healthcare)	Yes	1159	85
	No	205	15
Additional languages	One	606	44.4
	Two	394	28.9
	Three	139	10.2
	Four	21	1.5
	Five	5	0.4
	None	199	14.6
Leisure time abroad before teaching	Yes	905	66.3
	No	459	33.7
Study time abroad before teaching	Yes	374	27.4
	No	990	72.6
Work time abroad before teaching	Yes	300	22
	No	1064	78
Friends other countries/cultures	Yes	958	70.2
	No	406	29.8
Acquired factors			
ERASMUS+ Experience as a faculty	No	1116	81.8
	Yes, once	110	8.1
	Yes, twice	48	3.5
	Yes, three times	26	1.9
	Yes, more than three times	64	4.7
Total time in ERASMUS+	Less than a month	164	12.0
	From one to 3 months	50	3.7
	More than 3 months	35	2.6
Other experiences of internationalization	Yes	554	40.6
	No	810	59.4
Cultural competence level			
CAS level			
	Low	6	0.4
	Rather good	0	0.0
	Good	242	18.0
	Very good	1100	81.6
CCB level			
	Low	86	8.4
	Rather good	0	0.0
	Good	583	57.0
	Very good	353	34.5
CCA level			
	Low	6	0.4
	Rather good	0	0.0
	Good	242	18.0
	Very good	1100	81.6

### 3.2 Level of Cultural Competence of Nursing Faculty

The mean cultural competence (CCA) score of nursing faculty was 70.5 (SD 14.47; range 4–100; CV 21%).

Faculty had greater cultural awareness and sensitivity (CAS) (mean 83.7; SD 11.77; range 7.8–100; CV 14%) than cultural competence behaviors (CCB) (mean 60.7; SD 21.25; range 4.1–100; CV 35%).

### 3.3 Association of Background and Acquired Factors to Cultural Competence

A significant association was found between the CCA results and the language of the questionnaire (*F*_4,1356_ 9.02; *p* < 0.001), level of education (*F*_2,1356_ 9.96; *p* < 0.001), possession of a nursing degree (*F*_1,1356_ 57.8; *p* < 001), previous professional experience in a healthcare context (*F*_1,1356_ 60.2; *p* < 0.001), previous leisure stays abroad before working life as a lecturer (*F*_1,1356_ 8.26; *p* < 0.004), having friends from other countries or cultures (*F*_1,1356_ 23.7; *p* < 0.001), ERASMUS+ experience as a lecturer in higher education (*F*_4,1356_ 4.31; *p* < 0.001) and having other experiences of international activities as a faculty “at home” (*F*_1,1359_ 27.1; *p* < 0.001) (Table 3).

**TABLE 3:** Association of background and acquired factors to Cultural Competences.

Language													
CCA	Language	*n*	Mean	SD	CV%	Min	Q1	Median	Q3	Max	*F*_4;1356_	*p*	*ω* ^2^
CAS (0–100)	Spanish	933	85.5	10.86	13	7.8	80.5	87	92.2	100	28.97	< 0.**001**	0.08
	English	189	77.8	14.8	19	9.1	71.4	80.5	88.3	100			
	Italian	126	79.9	9.75	12	43	73.25	81.4	87.1	98.6			
	Portuguese	68	87.9	9.57	11	57	84.3	88.6	95.7	100			
	Turkish	45	76.7	9.96	13	52	70.1	77.9	83.1	97.4			
CCB (0–100)	Spanish	930	60	21.67	36	4.1	44.9	61.2	76.5	100	11.12	< 0.**001**	0.03
	English	185	57.2	22.17	39	6.1	42.9	58.2	73.5	100			
	Italian	126	60.1	16.62	28	15	48.2	60.7	68.8	96.4			
	Portuguese	68	72.6	18.34	25	39	57.1	75	87.23	100			
	Turkish	45	73.4	13.6	19	31	66.3	77.6	82.7	93.9			
CCA GLOBAL (0–100)	Spanish	935	71	14.41	20	8.6	61.7	72	81.7	100	9.02	< 0.**001**	0.02
	English	189	65.6	17.22	26	4	58.3	69.7	76	93.1			
	Italian	126	67.7	11.69	17	30	60.02	68.1	75.8	94			
	Portuguese	68	78.9	12.64	16	54	68.72	81.8	90.05	98.2			
	Turkish	45	74.9	9.83	13	46	71.4	76.6	82.3	92.6			

Gender													
CCA	Gender	*n*	Mean	SD	CV%	Min	Q1	Median	Q3	Max	*F*_2;1356_	*p*	*ω* ^2^
CAS (0–100)	Female	948	84.23	11.44	0.14	7.8	79.2	85.7	92.2	100	2.9	0.06	—
	Male	406	82.68	12.42	0.15	9.1	76.6	84.4	90.9	100			
	NA	7	79.51	14.15	0.18	57.1	73.35	77.9	87.45	100			
CCB (0–100)	Female	942	61.41	21.23	0.35	4.1	46.15	62.2	77.6	100	1.61	0.2	—
	Male	405	59.14	21.3	0.36	4.1	44.9	59.2	75.5	100			
	NA	7	60.5	20.38	0.34	25.5	53.55	59.2	71.45	88.8			
CCA GLOBAL (0–100)	Female	949	71.04	14.45	0.2	4.6	61.7	72.5	81.1	100	2.42	0.09	—
	Male	407	69.15	15.19	0.22	4	60	70.3	79.55	98.3			
	NA	7	68.73	9.02	0.13	57.1	62.85	69.1	73.45	82.3			

Religious community													
CAA	Religious community	*n*	Mean	SD	CV%	Min	Q1	Median	Q3	Max	*F*_5;1356_	*p*	*ω* ^2^
CAS (0–100)	Christian	83	83.25	11.41	0.14	19.5	77.9	85.7	90.9	100	8.06	< 0.**001**	0.03
	Muslim	76	76.02	10.46	0.14	51.9	68.8	77.9	82.45	97.4			
	Budist	80	80.34	7.5	0.09	74	74	77.9	84.4	91.4			
	Other	81	81.3	14.54	0.18	54.5	69.77	85	92.28	100			
	No	86	86.23	11.85	0.14	7.8	80.5	88.3	93.5	100			
	NA	82	82.43	15.28	0.19	19.5	78.08	85.7	90.68	100			
CCB (0–100)	Christian	60	60.19	21	0.35	4.1	45.9	61.2	75.25	100	4.16	< 0.**001**	0.01
	Muslim	73	72.51	15.63	0.22	25.5	63.3	77.6	82.7	93.9			
	Budist	69	69.4	15.27	0.22	49	57.1	78.6	79.6	82.7			
	Other	67	67.49	24.95	0.37	28.6	43.9	79.8	85.45	100			
	No	61	60.9	21.73	0.36	7.1	45.2	62.2	78.6	100			
	NA	54	53.75	24.26	0.45	14.3	32.63	53.4	77.32	92.9			
CCA GLOBAL (0–100)	Christian	70	69.97	14.41	0.21	8.6	61.05	70.9	80	98.9	1.86	**0.10**	—
	Muslim	74	74.07	11.07	0.15	45.1	70.3	76.6	81.45	92.6			
	Budist	74	74.16	9.75	0.13	60	69.1	76.6	80.6	84.5			
	Other	74	73.51	16.86	0.23	46.3	64	79.55	86.15	91.4			
	No	71	71.48	15.32	0.21	4	62.3	72.6	82.3	100			
	NA	66	66.16	17.69	0.27	16.6	54.85	68.1	81.3	90.9			

Level of studies													
CCA	Level of studies	*n*	Mean	SD	CV%	Min	Q1	Median	Q3	Max	F_2,1356_	*p*	*ω* ^2^
CAS (0–100)	Graduate/Bachelor	196	79.4	15.17	0.19	9.1	74	81.8	89.6	100	16.3	< 0.**001**	0.02
	Postgraduate	486	84.11	10.9	0.13	7.8	78.6	85.7	91.4	100			
	Doctorate	679	84.73	10.96	0.13	33.8	79.2	87	92.2	100			
CCB (0–100)	Graduate/Bachelor	193	57.02	20.69	0.36	5.1	42.9	57.1	71.4	100	5.2	< 0.**001**	—
	Postgraduate	487	62.72	19.17	0.31	11.2	50	62.2	77.65	100			
	Doctorate	674	60.35	22.67	0.38	4.1	44.9	63.3	77.6	100			
CCA GLOBAL (0–100)	Graduate/Bachelor	196	66.28	16.88	0.25	4	57.1	68.6	76.72	97.7	9.96	< 0.**001**	0.01
	Postgraduate	488	71.69	13.17	0.18	8.6	62.9	72	81.1	98.3			
	Doctorate	679	70.78	14.82	0.21	24	61.2	72.6	82.3	100			

Nursing degree													
CCA	Nursing degree	*n*	Mean	SD	CV%	Min	Q1	Median	Q3	Max	F_1;1356_	*p*	*ω* ^2^
CAS (0–100)	Yes	986	84.63	10.85	0.13	19.5	79.2	87	92.2	100	20.9	< 0.**001**	0.01
	No	375	81.39	13.64	0.17	7.8	77.1	84.4	89.6	100			
CCB (0–100)	Yes	984	63.03	20.52	0.33	4.1	49.77	64.3	78.85	100	43.47	< 0.**001**	0.03
	No	370	54.61	21.97	0.4	4.1	38.8	54.8	71.18	100			
CCA GLOBAL (0–100)	Yes	987	72.29	13.71	0.19	11.4	64	73.1	82.3	98.9	57.76	< 0.**001**	0.04
	No	376	65.67	16	0.24	4	56	66.7	76.6	100			

Previous experience													
CCA	Previous experience	*n*	Mean	SD	CV%	Min	Q1	Median	Q3	Max	*F*_1;1356_	*p*	*ω* ^2^
CAS (0–100)	Yes	1157	84.48	10.89	0.13	7.8	79.2	85.7	92.2	100	30.91	< 0.**001**	0.02
	No	204	79.56	15.24	0.19	9.1	74.22	83.1	89.6	100			
CCB (0–100)	Yes	1155	62.22	20.41	0.33	4.1	49	63.3	77.6	100	39.81	< 0.**001**	0.03
	No	199	52.07	23.91	0.46	4.1	31.6	52	70.4	100			
CCA GLOBAL (0–100)	Yes	1159	71.73	13.83	0.19	8.6	62.9	72.6	81.7	100	60.24	< 0.**001**	0.04
	No	204	63.26	17.08	0.27	4	51.27	64	74.9	94.3			

Extra languages													
CCA	Extra languages	*n*	Mean	SD	CV%	Min	Q1	Median	Q3	Max	F_5;1356_	*p*	*ω* ^2^
CAS (0–100)	One	604	83.68	11.38	0.14	9.1	77.9	85.7	92.2	100	0.45	0.82	—
	Two	394	83.45	12.77	0.15	7.8	77.9	85.7	92.2	100			
	Three	139	83.4	12.39	0.15	28.6	77.9	85.7	92.2	100			
	Four	21	86.4	9.46	0.11	57.1	84.3	88.3	91.4	98.6			
	Five	5	83.12	3.59	0.04	77.9	81.8	83.1	85.7	87.1			
	None	198	84.48	10.78	0.13	33.8	79.2	86.35	92.2	100			
CCB (0–100)	One	603	60.99	20.49	0.34	4.1	48.6	62.2	75.5	100	0.16	0.98	—
	Two	390	60.1	21.79	0.36	5.1	44.9	60.2	77.6	100			
	Three	137	60.94	23.63	0.39	6.1	41.8	62.2	82.7	100			
	Four	21	61.83	29.38	0.48	12.2	46.9	64.3	86.7	100			
	Five	5	66.02	32.75	0.5	12.2	60.2	78.6	82.7	96.4			
	None	198	60.75	19.62	0.32	6.1	45.9	61.2	75.38	100			
CCA GLOBAL (0–100)	One	606	70.54	14.09	0.2	4	62.3	72	80	98.9	0.34	0.89	—
	Two	394	69.91	15.57	0.22	4.6	61.1	70.9	81.7	100			
	Three	139	70.28	16.34	0.23	25.1	59.4	73.1	82.5	97.1			
	Four	21	72.61	18.93	0.26	33.1	65.1	74.9	88.6	97.7			
	Five	5	73.68	18.83	0.26	42.9	71.4	80.6	80.6	92.9			
	None	198	71.14	12.77	0.18	31.4	61.25	72	79.4	98.3			

Leisure stay													
CCA	Leisure Stay	*n*	Mean	SD	CV%	Min	Q1	Median	Q3	Max	*F*_1;1356_	*p*	*ω* ^2^
CAS (0–100)	Yes	904	84.96	10.32	0.12	19.5	79.2	87	92.2	100	29.49	< 0.**001**	0.02
	No	457	81.33	13.91	0.17	7.8	75.3	84.4	90.9	100			
CCB (0–100)	Yes	900	61.19	21.11	0.35	5.1	46.9	62.2	76.5	100	1.27	0.26	—
	No	454	59.81	21.52	0.36	4.1	44.9	60.7	77.6	100			
CCA GLOBAL (0–100)	Yes	905	71.27	14.11	0.2	11.4	61.9	72.5	81.1	98.9	8.26	< 0.**004**	—
	No	458	68.86	15.61	0.23	4	60.6	70.9	80	100			

Study stays													
CCA	Study stay	*n*	Mean	SD	CV%	Min	Q1	Median	Q3	Max	*F*_1;1356_	*p*	*ω* ^2^
CAS (0–100)	Yes	374	84.67	10.36	0.12	28.6	80	87	92.2	100	3.21	0.07	—
	No	987	83.39	12.25	0.15	7.8	77.9	85.7	92.2	100			
CCB (0–100)	Yes	372	60.15	22.06	0.37	6.1	44.9	61.4	77.6	100	0.38	0.54	—
	No	982	60.94	20.95	0.34	4.1	46.52	61.9	76.5	100			
CCA GLOBAL (0–100)	Yes	374	70.62	14.25	0.2	24	61.7	72	80.6	98.9	0.06	0.81	—
	No	989	70.4	14.84	0.21	4	61.1	72	81.1	100			

WORKING STAYS													
CCA	Work stay	*n*	Mean	SD	CV%	Min	Q1	Median	Q3	Max	*F*_1;1356_	*p*	*ω* ^2^
CAS (0–100)	Yes	300	84.48	11.07	0.13	28.6	79.2	87	92.2	100	1.5	0.22	—
	No	1061	83.53	11.96	0.14	7.8	77.9	85.7	92.2	100			
CCB (0–100)	Yes	297	59.47	22.74	0.38	7.1	43.9	61.6	75.5	100	1.34	0.25	—
	No	1057	61.08	20.81	0.34	4.1	46.9	61.6	77.6	100			
CCA GLOBAL (0–100)	Yes	300	70.06	15.35	0.22	25.1	60.6	72	80	98.9	0.28	0.59	—
	No	1063	70.57	14.48	0.21	4	61.7	72	81.1	100			

Friends from other countries/cultures													
CCA	Friends other culture	*n*	Mean	SD	CV%	Min	Q1	Median	Q3	Max	*F*_1;1356_	*p*	*ω* ^2^
CAS (0–100)	Yes	957	84.64	11.09	0.13	7.8	79.2	87	92.2	100	19.11	< 0.**001**	0.01
	No	404	81.61	13.01	0.16	9.1	76.6	83.1	90.9	100			
CCB (0–100)	Yes	954	62.1	21.39	0.34	5.1	48	63.3	79.6	100	13.51	< 0.**001**	—
	No	400	57.46	20.59	0.36	4.1	43.57	58.2	72.4	100			
CCA GLOBAL (0–100)	Yes	958	71.71	14.61	0.2	9.7	62.9	73.1	82.3	100	23.7	< 0.**001**	0.02
	No	405	67.51	14.42	0.21	4	58.9	68.6	76.6	97.7			

ERASMUS + stays													
CCA	ERASMUS + stay	*n*	Mean	SD	CV%	Min	Q1	Median	Q3	Max	*F*_4;1356_	*p*	*ω* ^2^
CAS (0–100)	No	1113	83.75	11.49	0.14	7.8	78.6	85.7	92.2	100	0.89	0.47	—
	Once	110	83.95	11.92	0.14	49.4	78.23	85.7	92.2	100			
	Twice	48	84.01	14.87	0.18	19.5	78.88	87.65	93.7	100			
	Three times	26	79.68	13.7	0.17	40.3	73.83	81.15	87	100			
	> Three times	64	84.65	12.84	0.15	45.5	77.9	87.7	93.83	100			
CCB (0–100)	No	1108	59.87	20.92	0.35	4.1	45.9	60.7	75.5	100	5.68	< 0.**001**	0.01
	Yes, once	109	60.35	21.88	0.36	10.2	43.8	57.1	79.6	100			
	Yes, twice	47	64.37	21.18	0.33	5.1	52.55	67.3	77.55	100			
	Yes, three times	26	63.93	24.3	0.38	14.3	49.52	68.85	82.18	100			
	Yes, more than three times	64	72.18	21.59	0.3	12.2	65.05	75.5	86.7	100			
CCA GLOBAL (0–100)	No	1115	69.98	14.39	0.21	4	61.1	71.4	80	100	4.31	< 0.**001**	—
	Yes, once	110	70.29	15.34	0.22	30.9	59.52	70.6	82.6	98.3			
	Yes, twice	48	72.19	17.22	0.24	11.4	64.82	75.4	81.55	98.2			
	Yes, three times	26	70.8	16.29	0.23	30.9	62.83	74.85	83.3	90.3			
	Yes, more than three times	64	77.61	14.11	0.18	33.1	70.3	80.6	87.4	97.1			

ERASMUS+duration													
CCA	ERASMUS Time	*n*	Mean	SD	CV%	Min	Q1	Median	Q3	Max	*F*_2,246_	*p*	*ω* ^2^
CAS (0–100)	< 1 month	164	84.39	11.91	0.14	40.3	77.9	85.7	93.5	100	0.7	0.5	—
	1–3 months	50	82.71	15.6	0.19	19.5	78.23	87.05	92.2	98.7			
	> 3 months	35	81.96	13.5	0.16	51.9	72.05	84.4	92.2	100			
CCB (0–100)	< 1 month	163	63.12	22.21	0.35	10.2	46.9	66.3	80.6	100	1.43	0.24	—
	1–3 months	49	69.16	23.11	0.33	5.1	58.2	73.5	85.7	100			
	> 3 months	35	65.79	21.69	0.33	14.3	57.1	67.3	81.65	100			
CCA GLOBAL (0–100)	< 1 month	164	72.12	15.09	0.21	30.9	63.85	73.4	83.4	98.2	0.38	0.68	—
	1–3 months	50	74.33	17.97	0.24	11.4	64.72	77.4	88.38	98.3			
	> 3 months	35	72.93	15.59	0.21	30.9	66	77.1	82.85	96.4			

Other experiences of internatitation “AT HOME”													
CCA		*n*	Mean	SD	CV%	Min	Q1	Median	Q3	Max	*F*_1;1359_	*p*	*ω* ^2^
CAS (0–100)	Yes	554	84.69	11.17	0.13	19.5	79.2	87	92.2	100	6.11	0.01	—
	No	807	83.09	12.13	0.15	7.8	77.9	85.7	90.9	100			
CCB (0–100)	Yes	551	64.2	22.01	0.34	4.1	49	67.3	82.15	100	25.2	< 0.001	0.02
	No	803	58.35	20.39	0.35	4.1	44.9	58.2	73.5	100			
CCA GLOBAL (0–100)	Yes	554	72.94	14.99	0.21	11.4	64	74.9	83.97	98.9	27.1	< 0.001	0.02
	No	809	68.76	14.21	0.21	4	60.4	70.2	78.3	100			

*Note:* Level of competence: < 25 low, 26–50 rather good, 51–75 good, > 75 very good.

Abbreviations: ω^2^, omega‐squared; CAS, Cultural Awareness and Sensitivity dimension; CCA, Cultural Competence Assessment; CCB, Cultural competence Behavior dimension; CV%, coefficient of variation in %; F, F‐Snedecor and degrees of freedom; Max, maximum; Min, minimum; Q1, first quartile; Q3, third quartile; SD, standard deviation.

The following variables were not correlated with the CCA scores: the age of the participants, their gender, the religious community they identify with, their years of experience as a faculty, speaking additional languages as well as their mother tongue, the number of years of experience as a health care provider, having previously spent time abroad studying or working before working life as a lecturer, or the length of time spent abroad as a lecturer in Higher Education (Table 3).

The exploratory multivariate linear regression showed that the variables of educational level, having a nursing degree, professional experience in a health care context, and having leisure stays abroad explain 7.8% of the variance in CAS. The variables of gender, years of professional experience in a health care context, and ERASMUS+ experience as a lecturer in higher education explain 5.1% of the variance in CCB. Finally, the variables of gender, the educational level, having a nursing degree, and an ERASMUS+ experience explain 5.9% of the variance in CCA (Table 4).

**TABLE 4:** Exploratory models of multivariate linear regression for CCA.

Model	Factors	Unstandardized	S.E	Standardized	*t*	*p*	95% CI	*R*	*R* ^2^%
**CAS**									
Backward model	(Intercept)	88.9	5.27		16.9	< 0.001	78.5 to 99.3	0.28	7.8%
	Level of studies	2.8	1.24	0.15	2.25	0.025	0.35 to 5.25		
	Nursing Grade	4.64	2.37	0.13	1.96	0.051	0.02 to 9.31		
	Healthcare experience	0.16	0.07	0.14	2.13	0.034	−0.01 to 0.31		
	Leisure stays	3.07	1.75	0.12	1.76	0.080	−0.37 to 6.52		
Forward model	(Intercept)	78.8	3.58		22.0	< 0.001	71.7 to 85.8	0.23	5.3%
	Level of studies	3.17	1.25	0.17	2.54	0.012	0.7 to 5.63		
	Healthcare experience	0.16	0.08	−0.14	−2.1	0.037	−99.6 to −0.31		
**CCB**									
Backward model	(Intercept)	63.6	5.79		11.0	1.734 × 10^−22^	52.2 to 75.0	0.23	5.1%
	Gender	6.57	3.29	−0.14	−2.0	0.047	0.09 to 13.1		
	Healthcare experience	0.241	0.14	0.12	1.7	0.091	−0.04 to 0.52		
	ERASMUS experience	2.33	1.17	0.14	2.0	0.047	0.03 to 4.6		
Forward model	(Intercept)	60.58	2.88		21.1	9.303 × 10^−54^	54.9 to 66.2	0.15	2.4%
	Teaching experience	0.29	0.13	0.15	2.3	0.025	0.04 to 0.55		
**CCA**									
Backward model	(Intercept)	73.12	6.71		10.9	< 0.001	59.0 to 86.4	0.24	5.9%
	Gender	3.89	2.21	−0.12	−1.8	0.08	−0.47 to 8.25		
	Level of studies	2.90	1.57	0.13	1.8	0.066	−0.19 to 5.99		
	Nursing Grade	5.41	3.00	−0.12	−1.8	0.073	−0.51 to 11.3		
	ERASMUS experience	1.42	0.78	0.12	1.8	0.071	−0.12 to 2.96		
Forward model	(Intercept)	81.2	3.55		22.9	< 0.001	74.2 to 88.2	0.14	2.0%
	Nursing Grade	6.12	3.04	−0.14	−2.0	0.05	0.13 to 12.1		

Abbreviations: 95% CI, 95% confidence interval; *R*, coefficient of correlation; *R* ^2^%, percentage of explained variance; S.E, standard error; *t*, *t*‐Student.

## 4 Discussion

The current study was conducted to assess the cultural competence of nursing faculty from 17 European countries. Two main findings are discussed in this section: (a) faculty exhibited a ‘good’ range of cultural competence, and (b) the cultural competence of faculty was associated with and influenced by their demographic profiles and experiences related to other cultures.

The average cultural competence in the sample of nursing faculty studied in the present study reached a level of ‘good’ Nevertheless, no previous studies were found measuring the cultural competence of nursing faculty using the same instrument. Others, like Baghdadi and Ismaile (2018) or Abou Hashish et al. (2020) who used the Cultural Diversity Questionnaire for Nurse Educators‐Revised (CDQNE‐R) to measure CC in nursing educators from US and Arabia Saudi, conclude that they had a moderate level. Haller (2018) reported a positive relationship between the cultural competence of nursing educators and their interaction with culturally diverse students.

In the current study, faculty scored higher in cultural awareness and sensitivity than in cultural competence behavior, which agrees with other authors who name cultural desire, awareness, and knowledge as prerequisites for having an ethno‐relative orientation towards cultural difference in order to successfully perform in cultural encounters (Gradellini et al. 2023; Racine et al. 2021). Abou Hashish et al. (2020) found that cultural awareness was the strongest predictor of overall cultural competence level. According to others (Abou Hashish et al. 2020; Rahimi et al. 2023; Sánchez‐Ojeda et al. 2018), educator training is needed to be able to convey and teach students the necessary cultural competences to adequately care for patients. An adequate level of competence in faculty should be reflected in the level of competence acquired by their students, which requires lecturers who are committed to and trained in cultural diversity (Sánchez‐Ojeda et al. 2018). The European study carried out by Antón‐Solanas, Huércanos‐Esparza, et al. 2021 on the perception and experience of nursing faculty in teaching cultural competence, points out that culturally competent nursing faculty should be able to teach within the cultural context of their students, being able to adapt to it.

When studying factors influencing cultural competence, published literature presents contradictory findings. Thus, for example, the study conducted and published by Burns (2020) identified individual and professional nursing features, such as experience, ongoing training in cultural competence, and proficiency in a foreign language, as predictive factors for the acquisition of cultural competence. Like Burns' findings, the present study also highlights previous professional experience and educational level as predictive factors for cultural competence acquisition. However, unlike Burns (2020), proficiency in a foreign language did not show a statistical association with the level of cultural competence in this research.

In the context of clinical practice, a recent study carried out in Austrian Intensive Care wards assessed the cultural competence of registered nurses and nursing students using the same CCA tool, albeit in a different setting, and also reported a moderate‐high level of cultural competence of nurses and students (Osmancevic et al. 2023). Age, educational level, cultural diversity training experience, and self‐perceived cultural competence were the factors that significantly influenced the level of cultural competence among participants. However, multilingual ability or migrant background was not a significant factor in this respect. Therefore, it seems that a higher educational level linked to cultural competence training during nursing education seems essential to ensure a culturally competent healthcare workforce.

The result of the current study shows that faculty with a higher level of education or who have a nursing degree (compared to those educators who are doctors, biologists, pharmacists, etc.) obtained a higher level of cultural competence. Cultural competence in nursing education depends significantly on the educators' approach and experiences (Kardas and Sahin 2023). It is important to distinguish between nursing faculty and those from non‐nursing backgrounds in this context. Nursing faculty members often integrate cultural competence from a practical, clinical perspective, deeply influenced by their direct experience in patient care. Their teaching is typically rooted in real‐world clinical situations, allowing them to contextualize theoretical concepts with tangible, culturally diverse examples (Campinha‐Bacote 2002). On the other hand, non‐nursing faculty members typically approach cultural competence from a broader, interdisciplinary framework, drawing from fields such as sociology, anthropology, or ethics. These perspectives can enhance the understanding of social and cultural determinants of health but may lack the practical focus necessary for students to fully grasp the challenges of applying these concepts in real healthcare settings. Their approach can offer valuable insights into systemic and global health issues but may not always translate easily into everyday clinical practice (Purnell 2014). Therefore, fostering collaboration between these two groups is crucial to maximize educational impact. Nursing students can acquire a well‐rounded education in cultural competence by developing integrated curricula that combine both theoretical and practical elements. This integrated approach will better prepare them to meet the complex, multicultural demands of modern healthcare delivery (Foronda et al. 2016).

Also, faculty who had friends from other countries or cultures and international experiences abroad (Erasmus +) or “at home” had a higher level of cultural competence. Although significant differences were identified when comparing CC to different factors, the effect found was small (except for the variables of being a nurse and having previous professional experience) which means that, even though these variables may have an influence on the competence score, their impact is low.

With regard to the educational level and previous working experience, the study published by Abou Hashish et al. (2020) points out that working experience and cultural competence training in the workplace constitute predictor variables that are related to an increased level of cultural competence. Nevertheless, Osmancevic et al. (2023) reported that educational level influences cultural competence more than work experience, with higher educational levels being linked to higher levels of cultural competence. According to Cicolini et al. (2015), this influence may be caused by the greater exposure to cultural diversity that usually occurs in higher education.

Concerning the influence of speaking other languages and participating in international activities, findings by Abou Hashish et al. (2020) point towards working experiences and speaking other languages as predictor factors for greater cultural competence in nursing faculty, while Baghdadi and Ismaile (2018) support languages spoken other than English and including cultural content in the current nursing program curriculum. Sargent et al. (2005) found that cultural competence levels of nurse educators are influenced by their knowledge of a foreign language, getting involved in exchange programs, and visiting a foreign country. International exchange programs constitute excellent opportunities for students to increase future nurses' preparedness for culturally competent practice, with systematic reviews focusing on identify and synthesize what kind of cultural competencies nursing students acquire while on these exchanges (Kokko 2011). Recent literature also shows that students have a significant increase in their cultural knowledge after undertaking international exchange programs with respect to their staying‐at‐home peers (Wang et al. 2021). We believe it is the same for educators. Nonetheless, not only have international exchanges been proved to enhance intercultural competencies, but also internationalization activities at home have a significant effect on cultural awareness among nursing students (Kin Kor et al. 2022) and nursing staff (Sung and Park 2021). Others, like Racine et al. (2021), raise some concerns about the usefulness of international stays when it comes to developing cultural competence and cultural safety, stating that international placements can be useful to raise consciousness about racial and social privileges, but can also involve some risks where participants reproduce harmful relations (i.e., perpetuating colonial and racialized power dynamics when they prioritize showcasing cultural, gendered, and racial differences as objects of study rather than fostering critical awareness of systemic privileges and inequities) (Racine et al. 2021).

Finally, the multivariate analysis in the present study showed that profiles of faculty with a Nursing Degree, professional experience, and leisure stays abroad show higher scores in the Cultural Awareness and Sensitivity scale by 7.8%. Profiles of women with professional experience and ERASMUS+ experience show higher scores in the Cultural Competence Behavior scale by 5.1%. In aggregate, women with a higher education, with a Nursing Degree, and ERASMUS+ experiences show higher scores in the Cultural Competence Assessment scale by 5.9%.

In the results of the current study, it stands out how several questions of the questionnaire did not pertain to faculty not teaching in the clinical setting. Examples of this can be found in some of the items of the Cultural Competence Behavior subscale that make explicit reference to interacting with patients (12 ‐“I include cultural assessment when I do patient or collective evaluation”, 16‐ “I ask patients and families to tell me about their explanations of health and illness”, 17‐ “I ask patients and families to tell me about their expectations for care”, 19‐ “I recognize potential barriers to services that might be encountered by different people”, 20‐ “I act to remove obstacles for people of different cultures when I identify such obstacles”…). Although this might not be relevant in the overall results, given that the percentage of faculty without healthcare experience is only 15% of the total, it does suggest that it would be necessary to adapt the questionnaire to be used among faculty who don't have direct contact with patients, since they also have a relevant role in the development of students' cultural competences.

### 4.1 Limitations

This study could be considered the first European study that assessed the cultural competence of nursing faculty. It aims to provide a deeper understanding of the factors affecting the cultural competence level of European faculty and recognize the training needs of nursing educators when it comes to cultural competence. Nevertheless, several limitations should be addressed. The study gathered a considerable sample size across multiple countries. However, participants were selected through consecutive sampling, and samples were only taken from institutions that were accessible to the researchers. These factors limit how broadly the findings can be applied. To improve generalizability, it is suggested that future studies involve larger sample sizes and include a wider range of institutions, capturing more diverse population groups within each country. Although the study relied on self‐reported data, the researchers implemented strategies to reduce potential bias. These included ensuring participants of the confidentiality of their data and identities, clearly explaining the study's purpose and significance, and providing reassurance that their involvement would not impact them negatively.

The CCA scale, despite having been translated and validated into several languages, does not include the same number of items in all of them, which does not allow for comparing results between the different countries participating in the project. Furthermore, it has never been used in the educational field, which explains why some items have not been answered by those educators not linked to healthcare practice. The results obtained offer the opportunity to improve the questionnaire so it can be used in the health teaching field. Additionally, as cultural competence achievement is an ongoing development process that involves the acquisition and integration of cultural awareness and cultural knowledge leading to cultural behavior (Campinha‐Bacote 2002), future researchers might consider conducting longitudinal studies in nursing faculty members after following specific educational programs. This would allow a better understanding of the development of cultural competence among them and help improve the cultural training of future nurses.

## 5 Conclusions

This research explored cultural competence among nursing faculty across various countries. The study revealed varying levels of cultural competence in the 17 nations examined; however, a common perspective emerged, generally rated as ‘good,’ with an emphasis on awareness and sensitivity rather than on behavior. The study identified several factors that influence cultural competence, such as gender, education level, holding a nursing degree, and having international experience.

## Clinical Resources

European Commission. Erasmus + (EU programme for education, training, youth and sports): https://erasmus‐plus.ec.europa.eu/.

MultiCulturalCare Project. Educating students through innovative learning methods to intervene in multicultural complex contexts: https://multiculturalcare.esenfc.pt/.

TraINErS‐ Project. An Intercultural Journey for Students, Teachers, and Health Care: https://trainers.ap.be/.

World Council on Intercultural and Global Competence: https://iccglobal.org/.

## Conflicts of Interest

The authors declare no conflicts of interest.

## Supporting information


Tables S1‐S2.

## Data Availability

The data that support the findings of this study are available from the corresponding author upon reasonable request.
