# Supplementary material for: Cultural Competence of European Nursing Faculty. An International Cross‐Sectional Study
Source: J Nurs Scholarsh. 2025 Mar 16;57(3):452–71. doi: 10.1111/jnu.70000 (PMC12064841; doi:10.1111/jnu.70000)
Supplement: Supplementary file 1 — Tables S1‐S2. [file JNU-57-452-s001.docx]

**Table 1 sup.** Participants (Language. Country and Higher Education Institution (n. %)

| **LANGUAGE CCA** | **COUNTRY** | **n** | **%** | **HIGHER EDUCATION INSTITUTION** | **n** | **%** |
| --- | --- | --- | --- | --- | --- | --- |
| **English** n=190 (13.9%) | Belgium | 18 | 1.3 | Belgium:Thomas More University of Applied Sciences -Campus Lier (TML) | 9 | 2.1 |
|  |  |  |  | Belgium:Thomas More University of Applied Sciences -Campus Turnhout (TMT) | 9 | 2.1 |
|  | Denmark | 38 | 2.8 | Denmark:University College Absalon (UCA) | 6 | 1.4 |
|  |  |  |  | Denmark:University College Copenhaguen (KP) | 32 | 7.5 |
|  | France | 13 | 1 | France:Institut de Formation en Soins Infirmiers du CHRU de Nancy (IFSI) | 12 | 2.8 |
|  | Germany | 3 | 0.2 | Germany:Frankfurt University of Applied Sciences (FRA-UAS) | 3 | 0.7 |
|  | Greece | 15 | 1.1 | Greece:University of Thessaly (UTH) | 16 | 3.7 |
|  | Holand | 9 | 0.7 | Holand: Hanzehogeschool Groningen (HUAS) | 9 | 2.1 |
|  | Ireland | 3 | 0.2 | Ireland:University College Cork (UCC) | 3 | 0.7 |
|  | Latvia | 7 | 0.5 | Latvia:Rīgas Stradiņa universitāte (RSU) | 7 | 1.6 |
|  | Malta | 19 | 1.4 | Malta:University of Malta (UoM) | 19 | 4.4 |
|  | Scotland | 8 | 0.6 | Scotland: Glasgow Caledonian University (GCU) | 8 | 1.9 |
|  | Slovakia | 39 | 2.9 | Slovakia: Slovak Medical University in Bratislava (SMU) | 39 | 9.1 |
|  | Sweden | 3 | 0.2 | Sweden: Linnaeus University (LU) | 3 | 0.7 |
|  | Switzerland | 15 | 1.1 | Switzerland: Bern University of Applied Sciences (BFH) | 15 | 3.5 |
| **Spanish** n=935 (68.5%) | Spain | 935 | 68.5 | Campus Docent Sant Joan de Deu (Barcelona) | 1 | 0.1 |
|  |  |  |  | Centro Universitario de Enfermería Salus Infirmorum (Cádiz) | 17 | 1.8 |
|  |  |  |  | Centro Universitario San Juan de Dios de Bormujos (Sevilla) | 9 | 1 |
|  |  |  |  | Centro Universitario San Rafael-Nebrija (Madrid) | 23 | 2.5 |
|  |  |  |  | Escuela Universitaria de Enfermería de Vitoria-Gasteiz (País Vasco) | 19 | 2 |
|  |  |  |  | Universidad Autónoma de Madrid (Cruz Roja) (Madrid) | 9 | 1 |
|  |  |  |  | Universidad Católica de Ávila (Cantabria) | 12 | 1.3 |
|  |  |  |  | Universidad Católica de Murcia (Murcia) | 44 | 4.7 |
|  |  |  |  | Universidad de Alcalá (Madrid) | 29 | 3.1 |
|  |  |  |  | Universidad de Barcelona (Barcelona) | 60 | 6.4 |
|  |  |  |  | Universidad de Burgos (Burgos) | 25 | 2.7 |
|  |  |  |  | Universidad de Castilla la Mancha (Albacete) | 12 | 1.3 |
|  |  |  |  | Universidad de Castilla la Mancha (Ciudad Real) | 6 | 0.6 |
|  |  |  |  | Universidad de Castilla la Mancha (Cuenca) | 11 | 1.2 |
|  |  |  |  | Universidad de Castilla la Mancha (Talavera) | 9 | 1 |
|  |  |  |  | Universidad de Castilla la Mancha (Toledo) | 16 | 1.7 |
|  |  |  |  | Universidad de Córdoba (Córdoba) | 10 | 1.1 |
|  |  |  |  | Universidad de Extremadura (Cáceres) | 5 | 0.5 |
|  |  |  |  | Universidad de Extremadura (Plasencia) | 9 | 1 |
|  |  |  |  | Universidad de Granada (Ceuta) | 19 | 2 |
|  |  |  |  | Universidad de Granada (Granada) | 50 | 5.3 |
|  |  |  |  | Universidad de Granada (Melilla) | 14 | 1.5 |
|  |  |  |  | Universidad de Jaén (Jaén) | 21 | 2.2 |
|  |  |  |  | Universidad de La Laguna (Tenerife) | 20 | 2.1 |
|  |  |  |  | Universidad de la Rioja (La Rioja) | 14 | 1.5 |
|  |  |  |  | Universidad de las Islas Baleares (Baleares) | 35 | 3.7 |
|  |  |  |  | Universidad de León (León) | 28 | 3 |
|  |  |  |  | Universidad de León (Ponferrada) | 11 | 1.2 |
|  |  |  |  | Universidad de Lérida (Lérida) | 9 | 1 |
|  |  |  |  | Universidad de Málaga (Málaga) | 21 | 2.2 |
|  |  |  |  | Universidad de Murcia (Murcia) | 40 | 4.3 |
|  |  |  |  | Universidad de Navarra (Navarra) | 16 | 1.7 |
|  |  |  |  | Universidad de Oviedo (Gijón) | 17 | 1.8 |
|  |  |  |  | Universidad de Oviedo (Oviedo) | 24 | 2.6 |
|  |  |  |  | Universidad de Santiago (Santiago) | 5 | 0.5 |
|  |  |  |  | Universidad de Valladolid (Valladolid) | 23 | 2.5 |
|  |  |  |  | Universidad de Vic | 16 | 1.7 |
|  |  |  |  | Universidad del País Vasco (Donosti) | 16 | 1.7 |
|  |  |  |  | Universidad del País Vasco (Leioa) | 26 | 2.8 |
|  |  |  |  | Universidad Europea de Madrid (Madrid) | 30 | 3.2 |
|  |  |  |  | Universidad Francisco de Vitoria (Madrid) | 11 | 1.2 |
|  |  |  |  | Universidad Jaume I de Castellón (Castellón) | 21 | 2.2 |
|  |  |  |  | Universidad Pontifica de Salamanca-Salus Infirmorum (Madrid) | 13 | 1.4 |
|  |  |  |  | Universidad Pontificia de Salamanca (Salamanca) | 32 | 3.4 |
|  |  |  |  | Universidad Rey Juan Carlos de Madrid (Madrid) | 27 | 2.9 |
|  |  |  |  | Universitat de Girona (Girona) | 29 | 3.1 |
|  |  |  |  | Universitat Rovira i Virigili (Tarragona) | 21 | 2.2 |
| **Italian** n= 127 (9.3%) | Italy | 127 | 9.3 | Italy:University of Verona. Campus Bolzano (UVB) | 5 | 1.2 |
|  |  |  |  | Italy:Centro Studi San Giovanni di Dio. Roma (Centro Studi FBF) | 9 | 2.1 |
|  |  |  |  | Italy:Humanitas (HUNIMED) | 57 | 13.3 |
| **Portuguese** n=67 (4.9%) | Portugal | 67 | 4.9 | Portugal: Escola Superior de Enfermagem de Lisboa (ESEL) | 24 | 5.6 |
|  |  |  |  | Portugal: Escola Superior de Enfermagem do Porto (ESENF) | 23 | 5.4 |
|  |  |  |  | Portugal: Escola Superior de Enfermagem San José de Cluny (ESESJCluny) | 11 | 2.6 |
|  |  |  |  | Portugal: Escola Superior de Saúde Fernando Pessoa: Porto (ESS-FP) | 10 | 2.3 |
| **Turkish** n=45 (3.3%) | Turkey | 45 | 3.3 | Turkey: Ege University Izmir (EUI) | 45 | 10.5 |

**Table 2 sup.** Frequencies for the CCA questionnaire items.

| **Item CCA questionaire** | **Frequency** | **Percent(%)** |
| --- | --- | --- |
| 1. **The race is the most important factor in determining a persons culture** | | |
| Strongly agree | 28 | 2.05 |
| Agree | 72 | 5.28 |
| Somewhat agree | 181 | 13.3 |
| Neutral | 99 | 7.26 |
| Somewhat disagree | 111 | 8.14 |
| Disagree | 267 | 19.6 |
| Strongly disagree | 572 | 41.9 |
| No opinion | 34 | 2.49 |
| Total | 1364 | 100 |
| 1. **People with a common cultural background think and act alike** | | |
| Strongly agree | 17 | 1.25 |
| Agree | 111 | 8.14 |
| Somewhat agree | 343 | 25.2 |
| Neutral | 126 | 9.24 |
| Somewhat disagree | 207 | 15.2 |
| Disagree | 283 | 20.8 |
| Strongly disagree | 260 | 19.1 |
| No opinion | 17 | 1.25 |
| Total | 1364 | 100 |
| 1. **Many aspects of culture influence health and healthcare** | | |
| Strongly disagree | 17 | 1.25 |
| Disagree | 12 | 0.88 |
| Somewhat disagree | 27 | 1.98 |
| Neutral | 52 | 3.81 |
| Somewhat agree | 206 | 15.1 |
| Agree | 497 | 36.4 |
| Strongly agree | 538 | 39.4 |
| No opinion | 15 | 1.1 |
| Total | 1364 | 100 |
| 1. **Aspects of cultural diversity need to be assessed for each individual. group. and organization** | | |
| Strongly disagree | 25 | 1.83 |
| Disagree | 18 | 1.32 |
| Somewhat disagree | 25 | 1.83 |
| Neutral | 98 | 7.18 |
| Somewhat agree | 151 | 11.1 |
| Agree | 472 | 34.6 |
| Strongly agree | 538 | 39.4 |
| No opinion | 37 | 2.71 |
| Total | 1364 | 100 |
| 1. **If I know about a person’s culture. I do not need to assess their personal preferences for health services** | | |
| Strongly agree | 25 | 1.83 |
| Agree | 33 | 2.42 |
| Somewhat agree | 45 | 3.3 |
| Neutral | 62 | 4.55 |
| Somewhat disagree | 102 | 7.48 |
| Disagree | 330 | 24.2 |
| Strongly disagree | 722 | 52.9 |
| No opinion | 45 | 3.3 |
| Total | 1364 | 100 |
| 1. **Spirituality and religious beliefs are important aspects of many cultural groups** | | |
| Strongly disagree | 14 | 1.03 |
| Disagree | 12 | 0.88 |
| Somewhat disagree | 17 | 1.25 |
| Neutral | 43 | 3.15 |
| Somewhat agree | 104 | 7.62 |
| Agree | 503 | 36.9 |
| Strongly agree | 658 | 48.2 |
| No opinion | 13 | 0.95 |
| Total | 1364 | 100 |
| 1. **Individuals may identify with more than one cultural group** | | |
| Strongly disagree | 12 | 0.88 |
| Disagree | 13 | 0.95 |
| Somewhat disagree | 18 | 1.32 |
| Neutral | 97 | 7.11 |
| Somewhat agree | 163 | 12.0 |
| Agree | 627 | 456.0. |
| Strongly agree | 413 | 30.3 |
| No opinion | 21 | 1.54 |
| Total | 1364 | 100 |
| 1. **Language barriers are the only difficulties for recent immigrants** | | |
| Strongly agree | 39 | 2.86 |
| Agree | 42 | 3.08 |
| Somewhat agree | 65 | 4.77 |
| Neutral | 40 | 2.93 |
| Somewhat disagree | 110 | 8.06 |
| Disagree | 355 | 26.0 |
| Strongly disagree | 683 | 50.1 |
| No opinion | 30 | 2.2 |
| Total | 1364 | 100 |
| 1. **I believe that everyone should be treated with respect no matter what their cultural heritage (no IT nor PT)** | | |
| Strongly agree | 15 | 1.28 |
| Agree | 3 | 0.26 |
| Somewhat agree | 5 | 0.43 |
| Neutral | 21 | 1.79 |
| Somewhat disagree | 10 | 0.85 |
| Disagree | 70 | 5.98 |
| Strongly disagree | 1036 | 88.6 |
| No opinion | 10 | 0.85 |
| Total | 1170 | 100 |
| 1. **I understand that people from different cultures may define the concept of healthcare in different ways** | | |
| Strongly disagree | 19 | 1.39 |
| Disagree | 12 | 0.88 |
| Somewhat disagree | 17 | 1.25 |
| Neutral | 43 | 3.15 |
| Somewhat agree | 112 | 8.21 |
| Agree | 479 | 35.1 |
| Strongly agree | 665 | 48.8 |
| No opinion | 17 | 1.25 |
| Total | 1364 | 100 |
| 1. **I think that knowing about different cultural groups helps direct my work with individuals. families. groups. and organization** | | |
| Strongly disagree | 20 | 1.47 |
| Disagree | 5 | 0.37 |
| Somewhat disagree | 6 | 0.44 |
| Neutral | 44 | 3.23 |
| Somewhat agree | 82 | 6.01 |
| Agree | 416 | 30.5 |
| Strongly agree | 775 | 56.8 |
| No opinion | 16 | 1.17 |
| Total | 1364 | 100 |
| 1. **I include cultural assessment when I do individual or collective evaluation** | | |
| Strongly disagree | 234 | 17.2 |
| Disagree | 165 | 12.1 |
| Somewhat disagree | 211 | 15.5 |
| Neutral | 126 | 9.24 |
| Somewhat agree | 161 | 11.8 |
| Agree | 220 | 16.1 |
| Strongly agree | 117 | 8.58 |
| No opinion | 130 | 9.53 |
| Total | 1364 | 100 |
| 1. **I seek information on cultural needs when I identify new people in my practice** | | |
| Strongly disagree | 150 | 11 |
| Disagree | 207 | 15.2 |
| Somewhat disagree | 264 | 19.4 |
| Neutral | 166 | 12.2 |
| Somewhat agree | 163 | 12.0 |
| Agree | 241 | 17.7 |
| Strongly agree | 112 | 8.21 |
| No opinion | 61 | 4.47 |
| Total | 1364 | 100 |
| 1. **I have resource webpages. books and other materials available to help me learn about people from different cultures** | | |
| Strongly disagree | 156 | 11.4 |
| Disagree | 229 | 16.8 |
| Somewhat disagree | 244 | 18.0 |
| Neutral | 172 | 12.6 |
| Somewhat agree | 152 | 11.1 |
| Agree | 233 | 17.1 |
| Strongly agree | 141 | 10.3 |
| No opinion | 37 | 2.71 |
| Total | 1364 | 100 |
| 1. **I use a variety of sources to learn about the cultural heritage of other people** | | |
| Strongly disagree | 150 | 11 |
| Disagree | 218 | 16.0 |
| Somewhat disagree | 267 | 19.6 |
| Neutral | 176 | 12.9 |
| Somewhat agree | 153 | 11.2 |
| Agree | 223 | 16.4 |
| Strongly agree | 137 | 10.0 |
| No opinion | 40 | 2.93 |
| Total | 1364 | 100 |
| 1. **I ask people to tell me about their explanations of health and illness** | | |
| Strongly disagree | 66 | 4.84 |
| Disagree | 130 | 9.53 |
| Somewhat disagree | 213 | 15.6 |
| Neutral | 188 | 13.8 |
| Somewhat agree | 182 | 13.3 |
| Agree | 311 | 22.8 |
| Strongly agree | 217 | 15.9 |
| No opinion | 57 | 4.18 |
| Total | 1364 | 100 |
| 1. **I ask people to tell me about their expectations for care** | | |
| Strongly disagree | 103 | 7.55 |
| Disagree | 130 | 9.53 |
| Somewhat disagree | 205 | 15.0 |
| Neutral | 160 | 11.7 |
| Somewhat agree | 189 | 13.9 |
| Agree | 297 | 21.8 |
| Strongly agree | 198 | 14.5 |
| No opinion | 82 | 6.01 |
| Total | 1364 | 100 |
| 1. **I avoid using generalizations to stereotype groups of people** | | |
| Strongly disagree | 47 | 3.45 |
| Disagree | 34 | 2.49 |
| Somewhat disagree | 71 | 5.21 |
| Neutral | 133 | 9.75 |
| Somewhat agree | 171 | 12.5 |
| Agree | 409 | 30.0 |
| Strongly agree | 472 | 34.6 |
| No opinion | 27 | 1.98 |
| Total | 1364 | 100 |
| 1. **I recognize potential barriers to education that might be encountered by different people** | | |
| Strongly disagree | 37 | 2.71 |
| Disagree | 62 | 4.55 |
| Somewhat disagree | 168 | 12.3 |
| Neutral | 169 | 12.4 |
| Somewhat agree | 247 | 18.1 |
| Agree | 409 | 30.0 |
| Strongly agree | 185 | 13.6 |
| No opinion | 87 | 6.38 |
| Total | 1364 | 100 |
| 1. **I act to remove obstacles for people of different cultures when I identify such obstacles** | | |
| Strongly disagree | 37 | 2.71 |
| Disagree | 77 | 5.65 |
| Somewhat disagree | 143 | 10.5 |
| Neutral | 229 | 16.8 |
| Somewhat agree | 234 | 17.2 |
| Agree | 364 | 26.7 |
| Strongly agree | 169 | 12.4 |
| No opinion | 111 | 8.14 |
| Total | 1364 | 100 |
| 1. **I act to remove obstacles for people of different cultures when others identify such obstacles to me** | | |
| Strongly disagree | 27 | 1.98 |
| Disagree | 61 | 4.47 |
| Somewhat disagree | 114 | 8.36 |
| Neutral | 201 | 14.7 |
| Somewhat agree | 203 | 14.9 |
| Agree | 410 | 30.1 |
| Strongly agree | 246 | 18.0 |
| No opinion | 102 | 7.48 |
| Total | 1364 | 100 |
| 1. **I welcome feedback from students about how I relate to others with different culture** | | |
| Strongly disagree | 37 | 2.71 |
| Disagree | 37 | 2.71 |
| Somewhat disagree | 81 | 5.94 |
| Neutral | 125 | 9.16 |
| Somewhat agree | 154 | 11.3 |
| Agree | 326 | 23.9 |
| Strongly agree | 443 | 32.4 |
| No opinion | 161 | 11.8 |
| Total | 1364 | 100 |
| 1. **I find ways to adapt my work to individual and collective cultural preferences** | | |
| Strongly disagree | 35 | 2.57 |
| Disagree | 76 | 5.57 |
| Somewhat disagree | 146 | 10.7 |
| Neutral | 176 | 12.9 |
| Somewhat agree | 252 | 18.5 |
| Agree | 372 | 27.3 |
| Strongly agree | 201 | 14.7 |
| No opinion | 106 | 7.77 |
| Total | 1364 | 100 |
| 1. **I document cultural assessments** | | |
| Strongly disagree | 186 | 13.6 |
| Disagree | 162 | 11.9 |
| Somewhat disagree | 172 | 12.6 |
| Neutral | 140 | 10.3 |
| Somewhat agree | 161 | 11.8 |
| Agree | 230 | 16.9 |
| Strongly agree | 124 | 9.09 |
| No opinion | 189 | 13.9 |
| Total | 1364 | 100 |
| 1. **I document the adaptations I make with students** | | |
| Strongly disagree | 167 | 12.2 |
| Disagree | 164 | 12.0 |
| Somewhat disagree | 177 | 13.0 |
| Neutral | 135 | 9.9 |
| Somewhat agree | 158 | 11.6 |
| Agree | 227 | 16.6 |
| Strongly agree | 150 | 11 |
| No opinion | 186 | 13.6 |
| Total | 1364 | 100 |
| 1. **I welcome comments from colleagues on how I relate to people from different cultures” (only in the Italian questionnaire** | | |
| Disagree | 1 | 0.79 |
| Somewhat disagree | 9 | 7.14 |
| Neutral | 42 | 33.3 |
| Somewhat agree | 14 | 11.1 |
| Agree | 29 | 23.0 |
| Strongly agree | 23 | 18.3 |
| No opinion | 8 | 6.35 |
| Total | 126 | 100 |
| 1. **I learn from my colleagues’ notions and information about cultural diversity” (only in the Italian questionnaire)** | | |
| Strongly disagree | 5 | 3.97 |
| Disagree | 7 | 5.56 |
| Somewhat disagree | 17 | 13.4 |
| Neutral | 24 | 19.1 |
| Somewhat agree | 20 | 15.9 |
| Agree | 35 | 27.8 |
| Strongly agree | 14 | 11.1 |
| No opinion | 4 | 3.17 |
| Total | 126 | 100 |
